# Hemorrhagic Diseases of the Kidney—Report of a Case of Probable Renal Varix

**Published:** 1912-06

**Authors:** Edgar G. Ballanger, Omar F. Elder

**Affiliations:** Atlanta, Ga.; Atlanta, Ga.


### HEMORRHAGIC DISEASES OF THE KIDNEY—REPORT OF A CASE OF PROBABLE RENAL VARIX.

By Edgar G. Ballenger, M. D., and Omar F. Elder, M. D., Atlanta, Ga.

With the advent of the cystoscope and ureteral catheterization has come such precision in the localization of pus and blood —two great sign boards pointing toward the disease, that we now operate with much less danger than when the diagnosis had to be made at the time of the operation. In practically no branch of medicine can more accurate diagnosis be made than in genito-urinary diseases, if the clinical history is supplemented with appropriate physical, chemical, microscopic, X-ray and cystoscopic examination. These preliminary examinations, and tests of the functional capacity of the kidneys have appreciably reduced the mortality of many bladder and kidney operations. The surgeon, too, derives much comfort from the knowledge of the important facts thus ascertained. In reality, the operation may be said to be more than half over when the necessary information has been carefully and accurately acquired.

In the present paper we will only refer to facts bearing upon renal hemorrhage as in the history to be given the bladder as the source of blood was definitely eliminated and a full discussion of blood in the urine would make the article longer than we wish it to be.

Having determined by cystoscopic examination or ureteral catheterization that blood comes from the right or left kidney, what are some of the diseases to be considered as likely or possible causes of hematuria? Undoubtedly a considerable percentage of the cases of painless hematuria of renal origin are due to acute or chronic nephritis. The acute may arise from the ingestion of poisons as cantharides, turpentine, etc., or as a result of certain infectious diseases. The urine is highly colored, scanty, albuminous, and contains casts, more or less blood and renal epithelium. Chronic parenchymatous nephritis may be attended with periodic hemorrhage, at times quite severe. The same may be said of chronic interstitial nephritis. The urinary findings and history, of course, indicate the probable cause of renal hemorrhage when nephritis produces it. We must not lose sight of the fact, however, that nephritis may be present with any other cause of bleeding from the kidney.

Tuberculosis may cause slight bleeding from the kidney but the history, pus in the urine and tubercle bacilli will serve to eliminate this cause.

Tumors of the kidney—benign and malignant—produce bloody urine and may be very difficult or impossible to diagnose except by the exploration of the kidney.

Stones in the kidney or ureter may be recognized by the history, X-ray examinations and wax-tipped ureteral catheters. Blood from a stone is usually slight in amount except after violent exercise or renal colic.

Severe apparently idiopathic hemorrhage from the kidney when none of the previously mentioned causes are present, is frequently due to *renal varix* or *localized angioma*. The hematuria seems nearly always to be unilateral and is painless except when clots block the ureter and cause renal colic.

Fenwick, McGowan, Whitney and Cabot and Pilcher have reported a number of instances where these dilated blood vessels on the papillae caused much hemorrhage. They are of chief interest because there is so little pathologic tissue present compared with the persistent and sometimes alarming haematuria.

This form of bleeding from the kidney is probably the kind often referred to by older writers as "essential" or "idiopathic" hematuria.

The following case history is clearly of this type and is of interest because of its existence for 16 years without being operated upon.

The patient was 52 years of age, tall and thin, his weight having fallen from 180 pounds to 130. His health gradually failed until he was compelled to discontinue all work. The haematuria was periodic and often profuse, the urine frequently being the color of port wine. The blood would cease as quickly as it appeared and seemed dependent upon no one factor, such as exercise, straining or riding in rough vehicles. There were dull aching neuralgic pains at times, in the right loin and at others quite severe renal colic; the pains then radiating down the ureter to the bladder and to the penis or right testicle. These intense colicky pains were apparently caused by the passage of blood clots through the ureter. Between attacks the urine was practically normal in quantity and quality. The heart and blood vessels were normal and the hemoglobin was 80% in spite of the great amount of the blood constantly, or rather intermittently, being lost through the urine. The patient spent one winter in Grady Hospital, in Atlanta, under medical treatment without receiving more than temporary benefit. His physical condition and decided nervousness had made it impossible for him to attend to business affairs. While the urine was blood red upon the day of the first ureteral catheterization, the bladder required comparatively little washing to obtain a sufficiently clear medium in which to work. This fact indicated the renal origin of the blood, as hemorrhage from the bladder more frequently renders cystoscopic examination difficult than does hemorrhage from the kidney. The ureters were catheterized and bloody urine found to come from the right and clear urine from the left side. An X-ray picture taken later showed no stone to be present, although the renal colic when clots were passing seemed significant. The ureters were catheterized a week later and bloody urine again came from the right and normal urine from the left kidney. Collargol injected into the pelvis of the right kidney and followed by an X-ray picture failed to show any pathologic condition. The physical examination was negative except for undue sensitiveness over the right kidney, which was normal in position. Thus we concluded that there was a tumor of the right kidney or a renal varix which caused the hematuria. We knew positively that the blood came from the right side and we also knew from renal functional tests, of which we have the highest regard for Rountree and Geraghty's phenosulphonephthalein, that the left kidney was in normal working condition.

An operation was advised and undertaken after a few days' preparatory treatment in the hospital, during which time the renal function was brought to its maximum. The kidney was easily reached though bound rather firmly with perirenal adhesions.

When delivered through the incision and carefully examined, nothing pathologic could be found. The cortex was then incised along Broedel's line and further search made for disease or stones, but neither could be found. Having positively determined, however, before the operation that the right kidney was bleeding and that the left was normal, we did a nephrectomy. While aware of the fact that nephrotomy is sometimes sufficient to stop hemorrhage of this character, it was deemed inexpedient at this time, on account of the prolonged history of hemorrhage and the necessity of stopping it at the earliest possible moment and in the most certain manner.

The hemorrhage ceased immediately and the patient made an uneventful recovery, the left kidney seeming to have had no difficulty in performing the double duty required of it.

Upon carefully cutting up the kidney nothing abnormal could be found except three small reddened areas.

The following report was submitted by Dr. Paullin, the pathologist to whom the kidney was sent for examination:

"The specimen consists of a kidney from which the capsule strips very easily.

"On section the cortical area appears about normal in thickness, and the markings are very clearly defined. In making numerous sections of the kidney one finds three small dark areas situated near the entrance of the pyramids into the pelvis of the kidney, varying in size from 1½ to 2 mm. in diameter. Because of the previous hardening of the specimen in formaldehyde, it is not possible to make out macroscopically a definite structure of these areas. Microscopically, however, two of them are found to be composed of extravasated blood, whereas the third is found to consist of numerous small, thin-walled, dilated blood vessels, bordering on the lower-most portion of the pyramid, supported by a thin, delicate framework of loose areolar tissue. The capillary walls are one cell in thickness, and the vessels are filled with red blood cells. In this loose areolar tissue one finds quite a number of extravasated red blood cells. The condition seems to be a varix, which had developed only partially within the pyramids, but extends to a certain extent outside the confines of this structure. The cortical portion of the specimen shows a very slight swelling of the cells lining the convoluted tubules. The glomeruli and other structures show nothing abnormal."

Respectfully submitted,

J. E. PAULLIN, M. D.

